# Mode of Action of Microbial Biological Control Agents Against Plant Diseases: Relevance Beyond Efficacy

**DOI:** 10.3389/fpls.2019.00845

**Published:** 2019-07-19

**Authors:** Jürgen Köhl, Rogier Kolnaar, Willem J. Ravensberg

**Affiliations:** ^1^Wageningen Plant Research, Wageningen University & Research, Wageningen, Netherlands; ^2^Linge Agroconsultancy B.V., Oosterhout, Netherlands; ^3^Koppert Biological Systems, Berkel en Rodenrijs, Netherlands

**Keywords:** biological control, plant diseases, mode of action, antagonist, risk assessment, screening

## Abstract

Microbial biological control agents (MBCAs) are applied to crops for biological control of plant pathogens where they act via a range of modes of action. Some MBCAs interact with plants by inducing resistance or priming plants without any direct interaction with the targeted pathogen. Other MBCAs act via nutrient competition or other mechanisms modulating the growth conditions for the pathogen. Antagonists acting through hyperparasitism and antibiosis are directly interfering with the pathogen. Such interactions are highly regulated cascades of metabolic events, often combining different modes of action. Compounds involved such as signaling compounds, enzymes and other interfering metabolites are produced *in situ* at low concentrations during interaction. The potential of microorganisms to produce such a compound *in vitro* does not necessarily correlate with their *in situ* antagonism. Understanding the mode of action of MBCAs is essential to achieve optimum disease control. Also understanding the mode of action is important to be able to characterize possible risks for humans or the environment and risks for resistance development against the MBCA. Preferences for certain modes of action for an envisaged application of a MBCA also have impact on the screening methods used to select new microbials. Screening of MBCAs in bioassays on plants or plant tissues has the advantage that MBCAs with multiple modes of action and their combinations potentially can be detected whereas simplified assays on nutrient media strongly bias the selection toward *in vitro* production of antimicrobial metabolites which may not be responsible for *in situ* antagonism. Risks assessments for MBCAs are relevant if they contain antimicrobial metabolites at effective concentration in the product. However, in most cases antimicrobial metabolites are produced by antagonists directly on the spot where the targeted organism is harmful. Such ubiquitous metabolites involved in natural, complex, highly regulated interactions between microbial cells and/or plants are not relevant for risk assessments. Currently, risks of microbial metabolites involved in antagonistic modes of action are often assessed similar to assessments of single molecule fungicides. The nature of the mode of action of antagonists requires a rethinking of data requirements for the registration of MBCAs.

## Introduction: Microbial Biological Control Agents

Biological control of plant diseases is the suppression of populations of plant pathogens by living organisms (Heimpel and Mills, 2017). Amongst beneficial microorganisms isolates can be selected which are highly effective against pathogens and can be multiplied on artificial media. Application of such selected and mass produced antagonists in high densities once or several times during a growing season is called “augmentative biological control” (Eilenberg et al., 2001; Heimpel and Mills, 2017; van Lenteren et al., 2018). For commercial augmentative biological control of diseases, growers use MBCAs containing living microorganisms, that are registered plant protection products produced by biocontrol companies. In some cases, antimicrobial metabolites produced by selected microbial organisms are included in the product, and some products even contain only antimicrobial metabolites without living cells of the antagonist (Glare et al., 2012). Legally speaking these compounds are considered chemical actives in the EU. Also mycoviruses and bacteriophages can be potential MBCAs against plant pathogens. In Australia, Brazil, Canada, Europe, Japan, New Zealand, and United States a total of 101 MBCAs has been registered in 2017 for disease control (van Lenteren et al., 2018).

**FIGURE 1:**
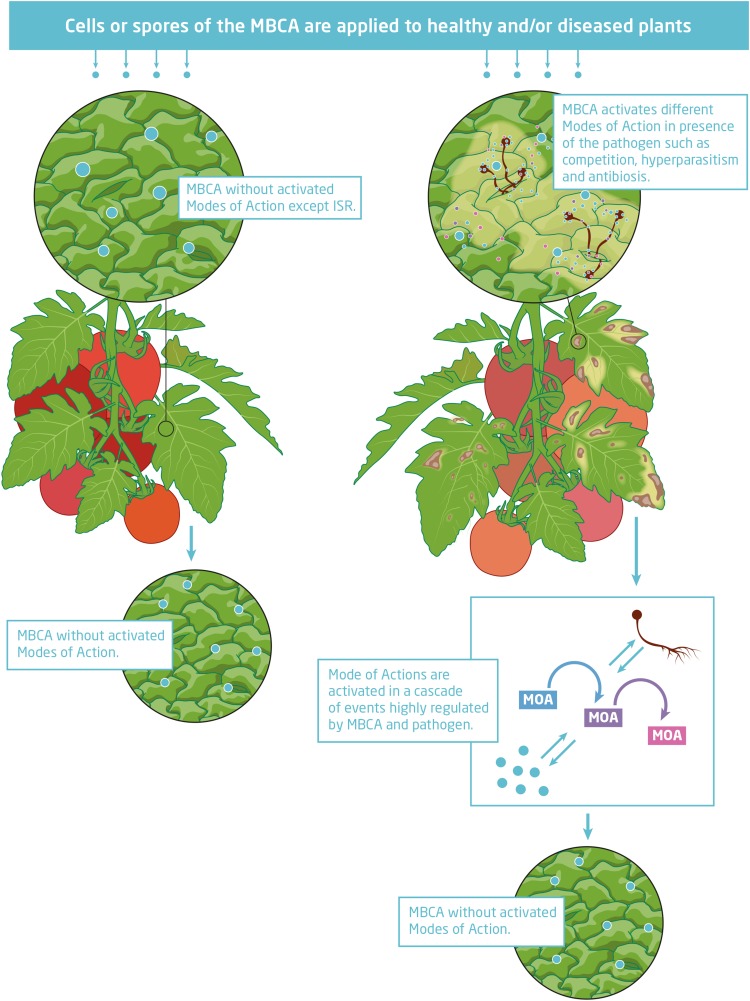
Microbial biological control agent (MBCA) temporally interacting *in situ* with the targeted pathogen activating different modes of action in cascades of events.

Microbial biological control agents protect crops from damage by diseases via different modes of action (Figure 1). They may induce resistance or prime enhanced resistance against infections by a pathogen in plant tissues without direct antagonistic interaction with the pathogen (Pieterse et al., 2014; Conrath et al., 2015). Another indirect interaction with pathogens is competition for nutrients and space (Spadaro and Droby, 2016). MBCAs may also interact directly with the pathogen by hyperparasitism or antibiosis. Hyperparasites invade and kill mycelium, spores, and resting structures of fungal pathogens and cells of bacterial pathogens (Ghorbanpour et al., 2018). Production of antimicrobial secondary metabolites with inhibiting effects against pathogens is another direct mode of action (Raaijmakers and Mazzola, 2012). Low amounts of *in situ* secreted secondary metabolites support antagonists to gain a competitive advantage. In some cases, biocontrol agents have been selected which secrete already efficient secondary metabolites into the growth media during mass production that are applied together with or without living cells of antagonists in the biological control product.

Pathogen populations thus can be limited by antagonistic microorganisms in very different ways. The nature of the mode(s) of action does not only determine how a pathogen population is affected by the antagonist. Also the characteristics of the MBCA depend on the exploited mode of action. Possible risks for humans or the environment, risks for resistance development against the biocontrol agent, its pathogen specificity and its dependency on environmental conditions and crop physiology may differ between different modes of action. Preferences for certain modes of action for an envisaged application of a biocontrol agent will also have impact on the screening methods used to select new antagonists (Köhl et al., 2011).

The objective of this paper is to review modes of action of microorganisms used to control plant diseases, building on recent detailed reviews on mechanisms of microorganisms antagonistic to plant pathogens (Compant et al., 2005; Lugtenberg and Kamilova, 2009; Raaijmakers and Mazzola, 2012; Pieterse et al., 2014; Conrath et al., 2015; Massart et al., 2015b; Spadaro and Droby, 2016; Karlsson et al., 2017; Mauch-Mani et al., 2017; Ghorbanpour et al., 2018; and various reviews on specific biocontrol microorganisms listed by Lugtenberg et al., 2013) with emphasis on implications for screening techniques, risk assessments and practical use. This review does not include biological control of invertebrates (van Lenteren et al., 2018) or weeds nor the incorporation of genes into antagonists or plants to enhance biocontrol efficiency and the risk assessment of such genetically modified organisms potentially used in biological control of plant diseases (Timms-Wilson et al., 2005).

## Interaction Via Plant Metabolism: Induced Resistance and Priming

Plants defend themselves with a broad variety of physical and chemical mechanisms against pathogens. Enhancing resistance is one of the most potential agronomic strategies to prevent biotic losses in crops. Constitutive mechanisms such as cuticles are complemented by inducible resistance mechanisms. Induced plant defense mechanisms are triggered by stimuli recognized by specific recognition receptors. Typical recognized stimuli are PAMPs which induce defense pathways in the plant to increase host resistance against the recognized attacking pathogen. Resistance can be induced locally in the attacked tissue or spread via signaling through the plant or even to neighboring plants resulting in SAR. This type of induced resistance is a direct reaction to the stimulus of necrotizing pathogens (Conrath et al., 2015). Another type of induced resistance is ISR where the enhanced defensive capacity of the whole plant to multiple pathogens is induced by beneficial microbes (Conrath et al., 2015). Both types of induced resistance decrease again in absence of the stimulus. Different to induction of resistance, priming of plants by stimuli leads to the sensitization for enhanced defense not only in the presence of the stimulus but also to a long lasting system of faster or stronger defense mechanisms in the future (Mauch-Mani et al., 2017). Even transgenerational priming has been reported (Conrath et al., 2015).

Resistance inducing stimuli produced by microorganisms are called MAMPs. These specific molecular signatures are recognized by corresponding extracellular PRRs. After signal reception early responding molecular mechanisms can be measured in the plant cells within a few minutes (Boller and Felix, 2009). The typical pathways of plants induced by MAMPs resulting in ISR have been studied in details and recently been reviewed by Pieterse et al. (2014). For priming, Conrath et al. (2015) and Mauch-Mani et al. (2017) recently reviewed compounds that are induced during the priming phase and molecular mechanisms that are present in plants in the post-challenge primed state.

Induced defense mechanisms involve the production of reactive oxygen species, phytoalexins, phenolic compounds, or pathogenesis-related proteins or the formation of physical barriers like modifications of cell walls and cuticles by the induced plant (Wiesel et al., 2014). These metabolic activities are energy-dependent so that long-lasting stimuli will result in energy costs for the plant to maintain induced defense mechanisms active. In contrast to directly induced resistance, priming of defense allows plants to react to stimuli later in a fast and robust manner with lower energy costs (Mauch-Mani et al., 2017). Stimuli inducing resistance and priming may be released from specifically selected MBCAs. However, plants are exposed also to stimuli from other origins, e.g., from pathogenic fungi or bacteria, herbivores, or abiotic stresses (Mauch-Mani et al., 2017). It is thus likely that crops grown in the field environment are frequently exposed to such stimuli inducing certain resistance levels so that applications of resistance inducing MBCAs may not result in additional induction of the resistance.

Plant defense reactions stimulated by MBCAs depend upon the plant genotype and are not considered in toxicological and eco-toxicological risk assessments of the MBCAs. Besides the general assessment of potential pathogenicity of the MBCAs for humans, animals, or plants, the released stimuli as acting mechanisms may be assessed for their toxicological and eco-toxicological risks. There is a broad range of potential MAMPs acting as stimuli, formerly called elicitors, belonging to very different groups of compounds (Boller and Felix, 2009). In their review, various pairs of MAMPS and the related PRRs are discussed but it is stated that many more MAMPs must exist in nature which have not been identified yet. The most studied MAMPs are the bacterial proteins flagellin and elongation factor EF-Tu. For both proteins the receptor systems are known and reactions are induced by subnanomolar concentrations. Other examples for MAMPs with known receptors are glucan, chitin, xylan, e.g., produced by *Phytophthora megasperma* and *Trichoderma viride*. Boller and Felix (2009) list many other MAMPs with still unknown receptors such as proteinaceous MAMPs from bacteria, e.g., superoxide dismutase and 23-amino-acid peptide, or from oomycetes, e.g., pep13 trans-glutaminase, sterol-binding elicitins, and cellulose-binding lectins; lipophylic MAMPs, e.g., ergosterol and arachidonic acid; oligosaccharide MAMPs, e.g., *N*-glycosylated yeast peptides, cell wall components from glucan and chitin and peptidoglycans; and lipooligosaccharides produced by gram-negative bacteria. Common to all signaling compounds is that they induce reactions in the plant cells when present at millimolar to subnanomolar levels (Boller and Felix, 2009).

Also siderophores produced by iron-competing bacteria (see below), antibiotics, such as DAPG and pyocyanin, biosurfactants and VOCs, such as 2R,3R-butanediol produced by *B. subtilis* GB03 (130) and a C13 volatile emitted by *Paenibacillus polymyxa* can act as elicitors inducing ISR (Pieterse et al., 2014). Mild viruses can also function as elicitors in plants. This principle is used for the biological control of Pepino mosaic virus in tomato which relies on mild variants of the Pepino mosaic virus (Schenk et al., 2010; European Food Safety Authority [EFSA], 2017). Products containing one or a combination of two mild virus isolates are commercially available.

There is very limited information on the background amounts of MAMPs in the environment or quantitative data on amounts produced by MBCAs after release in the environment. Persistent compounds that may be present at higher concentrations in the environment cannot have a signaling function in the interplay between microorganisms and plants that is strictly regulated in order to minimize resource expenditure and fine-tune signaling cascades (Mhlongo et al., 2018). It can thus be assumed that microorganisms including MBCAs release low amounts of such signaling compounds and that the compounds are exposed to rapid microbial and abiotic degradation processes so that concentrations in the environment generally are very low. It is expected that new approaches in “signalomics” based on metabolomics and metametabolomics will highlight the complex chemical communication within microbiomes and between microbiomes and plants including the contribution of signaling by released MBCAs to the continuous chemical cross-talk between organisms in the environment (Mhlongo et al., 2018).

There is no selection pressure on the pathogen via resistance inducing MBCAs themselves or by released signaling compounds because they do not interact directly with the pathogen. The development of resistance against induction of resistance is thus not likely (Romanazzi et al., 2016). However, the induced resistance itself causes selection pressure on the pathogen and pathogens may overcome induced resistance mechanisms (Bardin et al., 2015). This risk depends much on the evolutionary potential of the pathogen. Pathogens with mixed reproduction system, high potential for genotype flow, large effective population sizes and high mutation rates break plant resistance easier compared to pathogens with strict asexual reproduction, low potential for gene flow, small effective population sizes, and low mutation rates (McDonald and Linde, 2002).

Screening for MBCAs with high potential in induction of resistance is complex because the mode of action depends on a sequence of events from establishment of the MBCA, release of signaling compounds, induction of a cascade of metabolic events to induce plant defense mechanisms and the response of the pathogen to this defense mechanism. The final outcome of the biocontrol mechanism depends thus on the growing conditions for the MBCA on the plant, the physiology of the plant, the genetics of the chosen cultivar and the conditions for pathogen germination and infection. The final outcome of interactions of potential MBCAs with the plant and of the induced plant with the pathogen has to be quantified under standardized conditions, preferably under a range of environmental conditions and using different representative host genotypes. Screening systems can be simplified to allow high throughput screening by focusing in a first screening cycle on the selection of MAMPs with potential to induce known pathways in target host plants or even in cells of a model plant. For example, the expression of 28 genes involved in complementary plant defense mechanisms was measured in leaves of apple seedlings treated with potential resistance inducers (Dugé De Bernonville et al., 2014). Results correlated strongly with efficacies achieved with the tested inducers against *Erwinia amylovora* in trials under controlled conditions. In a comparable study, Badosa et al. (2017) used measurements of hydrogen peroxide production in tobacco cells in a first screening followed by measurements of expression of defense genes in tomato in a second screening. Selected inducers were effective in pear plants against *E. amylovora*.

In conclusion, using MBCAs for disease control through induction of resistance or priming relies on a complex sequence of events where the MBCA initially has to establish on the host, followed by the release of specific inducers which are recognized by specific receptors by the plant and thereafter triggering pathways within the host plant resulting in the onset of defense reactions or priming to make the plant ready for later challenges by pathogens. The first part of this sequence of events depends on the MBCA, the second part on the genetics and physiological status of the plant. MBCAs have to be selected in complex bioassays (Table 1) that are not standardized (Romanazzi et al., 2016). MAMPs released by the MBCA may be recognized by a specific plant species or a broader range of plant species so that one or several host pathogen combinations may be suppressed or controlled by the MBCA. The biocontrol effect depends also on the active colonization of the plant surface by the MBCA, which thus need appropriate ecological competence, and on the physiology of the plant, which needs the potential to develop a sufficiently high level of resistance. The MBCA induces defense reactions of the plant through a broad variety of signaling compounds which are low molecular weight compounds, in some cases breakdown products of cell walls. They are commonly produced by microorganisms at very low millimolar to subnanomolar concentrations for chemical communication between microorganisms and with plants. These processes are ubiquitous in the environment and common wherever different microorganisms coexist with plants. It can thus be assumed that the production of signaling compounds by applied MBCAs will pose very low toxicological or eco-toxicological risks (Table 2) and generally do not warrant a risk assessment.

**TABLE 1:** Modes of action in relation to development and use of microbial biological control agents.

**Mode of action**	**Method for screening**	**Pathogen specificity**	**Risk of resistance^1^**	**Dependency on environmental conditions**	**Dependency on plant physiology**	**Use by distributors and end user**
Induced resistance	Complex bioassay on plants	Specific to broad	Low	Low	High	Knowledge transfer needed
Competition	Simplified bioassays	Broad	Low	High	Low	Knowledge transfer needed
Hyperparasitism	Simplified bioassays	Pathogen specific interactions	Low	High	Low	Knowledge transfer needed
Antimicrobial metabolites produced *in situ*	Simplified bioassays	Specific to broad	Low	Moderate	Low	Knowledge transfer needed
Antimicrobial metabolites in product	*In vitro* assays	Broad	Moderate	Low	Low	Similar to use of fungicides
Helper strains^2^	Complex bioassays	Depends on MBCA	Low	Reduced	Reduced	Knowledge transfer needed
Assembled consortia combining different modes of action	*In silico* design followed by complex bioassays	Broad	Low	Low	Low	Knowledge transfer needed
Modulation of indigenous microbiota	Complex site-specific bioassays	Broad	Low	Medium	Low	Site-specific knowledge needed

*^1^Also depending on the specific evolutionary potential of targeted pathogen. ^2^Applied in combination with MBCAs.*

**TABLE 2:** Modes of action in relation to risk assessment and registration of microbial biological control agents.

**Mode of action**	**Risk of acute toxicity**	**Risks of metabolites**	**Environmental risks**	**Risks by environmental fate**	**Risks of phyto-toxicity**	**Analytical method**	**Recommended modification of current regulations**
Induced resistance	Very low	Very low	Very low	Very low	Low	Strain-specific	Simplification because of low intrinsic risks
Competition	Very low	Very low	Very low	Very low	Very low	Strain-specific	Simplification because of low intrinsic risks
Hyperparasitism	Very low	Very low	Very low	Very low	Very low	Strain-specific	Simplification because of low intrinsic risks
Antimicrobial metabolites produced *in situ*	Low	Low	Low	Low	Low	Strain-specific	Simplification because of low intrinsic risks
Antimicrobial metabolites in product	Risk assessment relevant	Risk assessment relevant	Risk assessment relevant	Risk assessment relevant	Risk assessment relevant	Metabolite-specific	Use current regulations for PPPs
Helper strains^1^	Low	Low	Low	Low	Low	Strain-specific	No registration required
Assembled consortia combining different modes of action	Low	Low	Low	Low	Low	Multiple strain-specific	New concept needed for overall risk assessments instead of risk assessment per active ingredient
Modulation of indigenous microbiota	Low	Low	Low	Low	Low	Microbiome characterization	No registration required

*^1^Applied in combination with MBCAs.*

## Indirect Interaction With Pathogens: Competition

Germination and growth of plant pathogens depend on nutrient uptake. Obligate biotrophic pathogens use exclusively nutrients from infected living host cells and do not depend on any exogenous nutrient sources in the environment outside the host plant (Agrios, 2005). The majority of plant pathogens exploit nutrient sources in a much less specific way by degrading dead organic plant matter. Necrotrophic plant pathogenic bacteria, fungi and oomycetes kill and subsequently invade tissues of host plants and utilize the available nutrients as primary colonizers of these killed tissues. Once necrosis has been induced by the pathogen, non-pathogenic microorganisms with saprophytic life style can potentially also colonize necrotic tissues so that a saprophytic competitive substrate colonization between different populations is common resulting in competition for nutrients and space. The principle competitive advantage of necrotrophic pathogens is that they are the first colonizers directly after they incited cell death. Non-pathogenic saprophytic endophytes being latently present in attacked host tissue may have a similar competitive advantage and may play an important role in competitive substrate colonization, e.g., of leaf lesions caused by necrotrophic pathogens.

The interaction with the host by killing and invading host tissue leading to damage in the infected crop is the most recognized stage of the life cycle of necrotrophic pathogens. However, most pathogen populations have another life style during significant parts of their life cycle when they live as saprophytes on necrotic plant tissues in soil, on crop residues, residues of non-hosts and on plant surfaces. During this stage pathogen populations survive, grow and spread independently of the host. The biology of such pathogens differs between species, some can develop completely independent from the host, other pathogen species will complete their life cycle only in the presence of the host during specific stages of their life cycle. Common for all necrotrophic pathogens during their saprophytic stage is that they depend on exogenous nutrients available in the environment, e.g., in colonized necrotic residues of host and non-host tissues. Successful host infection of most fungal necrotrophic pathogens also depends on exogenous nutrients during spore germination and formation of infection structures on host tissues (Chou and Preece, 1968; Fokkema et al., 1983). Also bacterial pathogens often depend on exogenous nutrients for multiplication to reach population levels sufficiently high to attack host tissues.

This dependency on exogenous nutrients during significant parts of their life cycle makes non-biotrophic pathogens vulnerable to nutrient competition (Köhl and Fokkema, 1998). Consequently, highly competitive microorganisms are potential candidates for biological control using competition for nutrients and space as mode of action. To exploit this mode of action in disease control, detailed knowledge on the epidemiology is essential to identify stages where limitations of nutrients and space will affect pathogen development. Typical situations are the presence of free nutrients in wounds of fruits which stimulate infection by various fruit pathogens, the presence of senescent floral tissues stimulating flower infection by *Botrytis cinerea* and the presence of dead host tissues on which the primary inoculum of pathogens is produced (Köhl et al., 1995; Calvo-Garrido et al., 2014; Spadaro and Droby, 2016). Potential competitive MBCAs must be able to occupy such niches, to survive and to consume rapidly nutrient sources essential for pathogen infection such as sugars, pollen and plant exudates on plant surfaces and in plant residues so that outcompeted pathogens will not be able to infect the host. The pathogen population will decline but will not be killed by the antagonist.

An example of use of this efficient mode of action is wound protection of fruits from pathogen invasion by fast colonizing yeasts (Spadaro and Droby, 2016). Yeasts as single cell organisms are able to multiply rapidly under favorable conditions in wounds of fruits which are rich in nutrient supply. They can consume a broad range of carbohydrates such as disaccharides and monosaccharides but also various nitrogen sources (Spadaro et al., 2010). Spadaro and Droby (2016) reviewed competition processes between antagonistic *Pichia guilliermondii* and pathogenic *Penicillium digitatum*, *P. expansum*, *B. cinerea*, or *Colletotrichum* spp. in wounds of different fruits and *Aureobasidium pullulans* and *P. expansum* in apple wounds. Competition for carbohydrates in the carbohydrate rich wound environment in combination with competition for the limited amounts of nitrogen sources such as amino acids play the key roles in the antagonistic interactions (for references: see Spadaro and Droby, 2016).

Besides carbohydrates and nitrogen sources, restricted iron availability due to the low solubility of Fe^3+^ ions can be a limiting factor for microbial growth. Many microorganisms can produce a variety of low-molecular-weight siderophores with a high affinity for ferric iron (van Loon, 2000). Microbial strains with the ability to produce high amounts of siderophores with high affinity to iron play an important role in disease suppression and can be selected for biological control through competition for iron with pathogens that produce lesser amounts of siderophores with lower affinity for iron (Bakker et al., 1993; van Loon, 2000; Whipps, 2001; Lugtenberg and Kamilova, 2009). This mechanism has been investigated in particular for isolates of *Pseudomonas* spp. and it has been demonstrated that siderophore mediated iron competition result in reduced pathogen populations in rhizospheres (Raaijmakers et al., 1995). Iron competition is also the mode of action of several fungal antagonists. For example, *Trichoderma asperellum* producing iron-binding siderophores controls *Fusarium* wilt (Segarra et al., 2010). The yeast *Metschnikowia pulcherrima* transforms pulcherriminic acid and iron ions to the red pigment pulcherrimin. This process leads to iron depletion in media inhibiting development of *B. cinerea*, *A. alternata*, and *P. expansum* (Saravanakumar et al., 2008).

Microbial biological control agents can also be targeted at the saprophytic stage of necrotrophic pathogens to outcompete the pathogen so that primary inoculum production on necrotic plant tissues is reduced or infections routes via senesced tissues to healthy tissues are blocked (Köhl and Fokkema, 1998). Examples are the application of *Microsphaeropsis ochracea* controlling *Venturia inaequalis* in apple (Carisse et al., 2000), *Clonostachys rosea* controlling *B. cinerea* in roses (Morandi et al., 2003), and *Ulocladium atrum* controlling *Botrytis* spp. in various crops. In cyclamen, colonization of senesced leaves by the pathogen is an essential step toward mycelial infections of attached healthy tissues. The antagonist can outcompete *B. cinerea* on senesced cyclamen leaves so that this infection route is blocked, resulting in disease control efficacy as obtained with conventional fungicides (Köhl et al., 1998). Resource capture by antagonists with resource competition as sole mechanism depends very much on the level of available nutrients and timing and distribution of the antagonist at the starting point of the interaction with the pathogen. A spatially explicit model has been developed by Kessel et al. (2005) which describes spatial and temporal competitive substrate colonization by *U. atrum* and *B. cinerea* under different simulated conditions. Such models can be applied to better understand effects of timing, densities and distributions on the outcome of competitive substrate colonization and thus to optimize biological control.

Competitive antagonists are usually screened for efficacy in bioassay systems under controlled conditions, e.g., on wounded fruit, seedlings, or necrotic host tissues. Since rapid growth and substrate colonization are of key importance during competition, these assays should be completed with screening assays for selection of antagonist with superior ecological competence (Köhl et al., 2011). If the most limiting nutrient source is already known for the envisaged antagonist, more simplified high throughput systems may be applied, e.g., competition assays with candidate antagonists and pathogen on nutrient media with limiting amounts of the identified nutrient source. Several methods have been applied to understand better the underlying processes during nutrient competition. Phenotypic microarray plates with different carbon and nitrogen sources can give first insights in the potential for competition between pathogen and antagonist using a nutritional similarity index. To unravel the mode of action of *A. pullulans* strains against *Monilinia laxa* in wound protection of peaches, Di Francesco et al. (2017) incubated *A. pullulans* and *M. laxa* in *in vitro* assays in peach juice. HPLC analysis of the growth medium revealed that specifically depletion of asparagine as nitrogen source restricts growth of *M. laxa*. In similar studies with a different strain of *A. pullulans* selected for protection of apple fruit from *P. expansum*, Janisiewicz et al. (2000) found that depletion of aspartic acid serine and glutamic acid in apple juice restricted the pathogen. Filonow (1998) used radiolabeled glucose to study its utilization by antagonistic yeasts and *B. cinerea*.

Competitive antagonists may modulate growth conditions for the pathogen in the targeted niche not only through nutrient depletion but also by other mechanisms. Application of *Bacillus brevis* resulted in fast drying of leaf surfaces and reduced *B. cinerea* by 68% similar to the application of a standard fungicide in Chinese cabbage (Seddon and Edwards, 1993). Modulating leaf wetness periods by antagonists, e.g., via secretion of biosurfactants, may be a powerful mode of action for prevention of leaf diseases and diseases in stored products without any direct interaction between pathogen and antagonist. *A. pullulans* strains antagonistic to *Erwinia amylovora* causing fire blight in pome fruit strongly reduce growth of the bacterial pathogen through shifting the pH of the medium down to pH 4.0 (Kunz, 2006). Acidifying of the growth substrate may be an additional mode of action supporting antagonists during competition with bacterial pathogens.

Pathogen populations are continuously exposed to environmental stresses such as extreme temperatures, drought, limiting nutrient availability, and sub-optimal pH values so that they are selected and adapted to environmental stresses common in their micro-habitat. Modulations of environmental stresses resulting in reduced nutrient availability by highly competitive MBCAs or in moderate changes of the pH or shortening of leaf wetness periods as demonstrated for *A. pullulans* (Kunz, 2006) and *Bacillus brevis* (Seddon and Edwards, 1993), respectively, will not add significant additional selection pressure on pathogen populations so that a build-up of resistance cannot be expected. Biological control using nutrient competition as mode of action works through the local and temporal increase of highly competitive biocontrol strains during defined critical development stages of the pathogens life cycles. Applied antagonists modulate growth conditions in the targeted niche making condition less favorable for pathogen development without any direct interaction with the pathogen. The antagonists produce enzymes to degrade complex organic matter, simple carbohydrates or amino acids or produce siderophores in case of competition for iron. These principle processes are basic for the ubiquitous saprophytic activities of microorganisms during utilization and decomposition of organic matter of plants or microorganisms, which are the fundamental processes to maintain nutrient cycling and plant growth in ecosystems. Different from plant pathogens, saprophytic fungi cannot colonize whole plants or fruits abundantly or cause spoilage. Consequently, it can be concluded that exploiting such common and essential processes in biological control will not cause environmental risks.

In conclusion, antagonists with nutrient competition as mode of action can be selected using adequate bioassays (Table 1). They often have a broader host range since modulation of environmental conditions in a micro-niche potentially affects various less competitive pathogens. Active metabolism and growth are essential for niche colonization and nutrient depletion. Thus, the efficacy of MBCAs strongly depend on their ecological competences. The risk for development of resistance against competition by pathogens can be considered as very low. Nutrient competition acts through enzyme activities and other mechanisms to bind limiting nutrients. Since these processes are ubiquitous in the environment and common wherever saprophytic microorganisms competitively colonize micro-niches, toxicological and eco-toxicological risks of adding nutrient competing antagonists to ecosystems can be considered as very low (Table 2), and do not generally warrant a risk assessment.

## Direct Interaction With Pathogens

### Hyperparasitism

Parasitism is the direct competitive interaction between two organisms in which one organism is gaining nutrients from the other. If the host is also a parasite, e.g., a plant pathogen, the interaction is defined as hyperparasitism. This kind of interaction is often observed between fungi. For bacteria, hyperparasitism rarely has been reported. *Bdellovibrio bacteriovorus* is a predatory bacterium which has the unusual property to use cytoplasm of other Gram-negative bacteria as nutrients (McNeely et al., 2017). In initial research on biological control, *B. bacteriovorus* was tested in liquid cocultures with phytopathogenic bacteria belonging to the *Burkholderia cepacia* complex. Specific strains of *B. bacteriovorus* predated a broad host panel of the pathogen complex. Predation by *B. bacteriovorus* strains of other plant pathogenic bacteria such as *Agrobacterium tumefaciens*, *Xanthomonas vesicatoria*, *X. campestris* pv. *campestris*, *Erwinia carotovora* pv. *carotovora*, *Pseudomonas syringae* pv. *glycinea*, *P. syringae* pv. *tomato*, *P. marginalis*, and *Erwinia herbicola* was confirmed in similar tests (McNeely et al., 2017).

In biotrophic mycoparasitism, the hyperparasite depends on the living host fungus and gains nutrients from the host cells via haustoria without killing the host. Host and mycoparasitic fungus interact in a stable and balanced way (Jeffries, 1995). These often species–specific interactions may be important components in disease suppressiveness in ecosystems but are hardly to be exploited for commercial augmentative biocontrol because mass production of the hyperparasite depends on living host mycelium as substrate. Hyperparasites with a necrotrophic life style gain nutrients from dead host cells but also from other commonly available organic matter which allow mass production on artificial media making this group of hyperparasites much more favorable for commercial use as MBCA compared to biotrophic hyperparasites. Necrotrophic hyperparasites invade host spores or hyphal cells after killing such cells. Main mechanisms of parasitism is the excretion of CWDEs combined in some cases with excretion of secondary metabolites in close contact with the host cell leading to openings in the cell wall and subsequent disorganization of the cytoplasm. Cell wall degradation is typically caused by a range of chitinases, β-1,3-glucanases and proteases or, in case of hyperparasites of oomycota, cellulases. Such a necrotrophic hyperparasitism with invasion of killed host cells is frequently observed by microscopy and electron microscopy. The assumed nutrient transfer from the dead host cell to the invading fungus often has not been proven because it is technically difficult to investigate such processes, especially in the *in situ* situation in the field (Jeffries, 1995).

For some pathogen groups, researchers thoroughly investigated the phenomenon of hyperparasitism and found many antagonistic fungal species. For example, 30 hyperparasitic species against *Rhizoctonia solani* belonging to 16 genera have been reported by Jeffries (1995). Obligate biotrophic pathogens have been of particular interest for biocontrol using hyperparasites. Hijmegen and Buchenauer (1984) report on eight hyperparasitic species of powdery mildews. Zheng et al. (2017) report on approximately 30 fungal species which show hyperparasitism against rust pathogens, including *Cladosporium uredinicola* against *Puccinia violae* (Traquair et al., 1984) and *Alternaria alternata* against *Puccinia striiformis* f. sp. *tritici* (Zheng et al., 2017). In bioassays on rust-inoculated wheat seedlings, *A. alternata* germ tubes contacted with and penetrated into urediniospores of the pathogen at 24 hpi, and caused complete urediniospore collapse at 36–48 hpi.

The most studied mycoparasites are belonging to the genera *Trichoderma* and *Clonostachys*. Antagonistic isolates of these genera vary in host range and individual strains mostly have a range of plant pathogenic hosts. They produce structures for attachment and infection, and kill their hosts by CWDEs, often in combination with antimicrobial secondary metabolites (Harman et al., 2004; Harman, 2006; Mukherjee et al., 2012; Karlsson et al., 2017; Nygren et al., 2018). These lytic enzymes are not constitutive but their production is triggered by complex signaling after recognition of the host. Surface compounds such as lectins from the host cell wall, surface properties and diffusible host-released secondary metabolites play important roles in the recognition and signaling pathways such as MAPK cascades, cAMP pathway and G-protein signaling (Karlsson et al., 2017). Recognition of the fungal host then leads to transcriptional reprogramming and expression of the “molecular weapons” involved in host attack and lysis, including certain CWDEs. Mycoparasitism-related gene families in *Trichoderma* such as ech42 and prb1 are upregulated during mycoparasitism. As result of the initial activities of CWDEs, oligosaccharides and oligopeptides are released by the host that are then recognized by *Trichoderma* receptors and thereby act as inducers (Karlsson et al., 2017). This attack by a necrotrophic mycoparasite results in further increase of permeability and degradation of host cell walls and death of the host. A synergistic transcription of various genes involved in cell wall degradation was also reported for *Trichoderma atroviride* in interaction with *B. cinerea* and *Phytophthora capsici* (Reithner et al., 2011).

Screening for hyperparasitic strains often is done by using host structures as baits, especially if such structures are large for easy handling and observations. Sclerotia, e.g., of *Sclerotinia sclerotiorum*, microsclerotia, e.g., of *R. solani*, individual urediniospores or pustules of rust pathogens, and individual conidia or pustules of powdery mildew have been exposed to potential antagonist candidates and macroscopical and microscopical observations were made to find strains which invade the host structures, often accompanied with discoloration of these structures. Such studies generally are completed by assessments of the viability of invaded host structures, e.g., Zheng et al. (2017) confirmed that the viability of urediniospores from *A. alternata* treated pustules was only 25% whereas 80% of spores from untreated rust pustules were viable.

Alternative screening of candidate antagonists for their activity of fungal CWDEs under *in vitro* conditions seems to be less adequate because activity levels of single enzymes in situations without interaction with the hosts will not be representative for the highly regulated interplay between antagonist and pathogen. In these interactions, different enzymes are secreted in subsequent events, regulated by signaling by different secondary metabolites (Karlsson et al., 2017). Furthermore, isolates selected for high constitutive enzyme production may not be strong competitors in competitive environments because they continuously invest into formation of metabolites which are needed only for their function in the particular situations of antagonism in close contact with the host. Due to this high complexity of a hyperparasitism, which often is a cascade of events, all depending on each other and leading to ultimate cell death only after activating the whole cascade, screening assays should not focus in a simplified way on single events, such as formation of a single enzyme, but should measure the final results of the entire cascade of events.

Enzymes such as CWDEs are complex proteins consisting of several 100 or 1000 amino acids with the function to catalyze the conversion of specific substrates into specific products. Functioning of enzymes depends not only on amino acid sequences but also on their complex tertiary structures (Iyer and Ananthanarayan, 2008). Unfolding of these structure or disordered polypeptides lead to enzyme denaturation and irreversible loss of the enzymatic activity. Enzymes are sensitive to physical denaturation, e.g., by heat or cold temperatures, chemical denaturation by various factors from acids to chelating agents and to microbial denaturation, e.g., by proteases. The generally high sensitivity of enzymes to denaturation is a main obstacle in technological processes so that enzyme stabilization during production and application is common in technological applications. Proteases, cellulases, lipases, amylases, and other enzymes are produced at industrial scales by microorganisms and are commonly used in paper processing, food manufacture, medical device cleaning, ethanol manufacture, as well as many common household cleaning processes such as laundry and dishwashing (Anonymous, 2002). Enzymes used for such technical applications have been tested through many years and it has been proven that enzymes have a very safe toxicological profile with a good record of occupational health and safety for the consumer. Studies revealed that enzymes seem unlikely to be dangerous to the aquatic environment due to their ready biodegradability and the low effects on aquatic organisms observed (Anonymous, 2002).

Cell wall-degrading enzymes are commonly produced in the environment by microorganisms during decomposition of organic matter originating from dead plant tissues and dead microorganisms including dead fungal hyphae, and continuously play an essential role in nutrient cycling in all ecosystems. Given this background activity of enzymatic CWDEs in natural ecosystems, application of hyperparasites in biological control will not significantly increase cell wall degrading activities in the environment. Hyperparasites produce low amounts of fungal CWDEs during short time periods locally in micro-niches when they interact with their hosts. The produced low amounts of chitinases, β-1,3-glucanases and proteases present in the environment very locally during short time periods are substrate-specific and highly sensitive to denaturation in the environment with its usually high microbial activity combined with chemical and physical factors enhancing enzyme denaturation. In conclusions relevant toxicological and ecotoxicological risks of hyperparasite applications can be considered as very low because activities are highly specific, production is restricted in time and space and rapid denaturation is common.

The development of resistance by a plant pathogen against hyperparasitism by a biological control agents has not yet been reported. Pathogens can develop resting structures such as endospores, chlamydospores, and melanised sclerotia with high resistance against hyperparasitism by naturally occurring antagonistic microorganisms (Bardin et al., 2015). Pathogens can also repress synthesis of enzymes needed by the antagonist for hyperparasitic interactions. A considerable variation in susceptibility of *S. sclerotiorum* to the commercially applied hyperparasite *Coniothyrium minitans* has been observed in different regions in France (Nicot et al., 2016). Sclerotia produced by the various strains of *S. sclerotiorum* differed in average thickness and thickness of their melanised cortical tissue. However, both morphological traits did not correlate with susceptibility to hyperparasitism by *C. minitans* (Nicot et al., 2018). With the background of continuous selection pressure by hyperparasites present in the natural microbiome it is not likely that a temporal increase of this pressure by an antagonist application will enhance resistance of the pathogen.

In conclusion, antagonists with hyperparasitism as mode of action can be selected using adequate bioassays (Table 1). They generally have a narrow host range and their activity depends on environmental conditions because their antagonistic activity depends on active growth. The risk for development of resistance against hyperparasites by pathogens can be considered as low. Hyperparasitism acts through CWDEs which production is highly regulated by signaling from the potential host pathogen. Since these enzymes, ubiquitously produced in all ecosystems, are highly substrate specific and highly susceptible to rapid degradation, toxicological and eco-toxicological risks can be considered as very low (Table 2) and do not warrant a risk assessment.

### Antibiosis by Antimicrobial Metabolites

Antimicrobial metabolites are secondary metabolites belonging to heterogeneous groups of organic, low-molecular weight compounds produced by microorganisms that are deleterious to the growth or metabolic activities of other microorganisms (Thomashow et al., 1997). They are produced and released to the environment in small quantities by many microorganisms. Huge numbers of known antibiotics are produced by actinomycetes (8700 different antibiotics), bacteria (2900) and fungi (4900) (Bérdy, 2005). Less than 1% of microscopically counted bacteria can be cultured on usual culture media (Amann et al., 1995). Approximately one-third of the bacterial divisions have no cultured representatives and are known only through rRNA sequences (Clardy et al., 2006). It thus can be assumed that the majority of antibiotics produced *in situ* in the environment is still unknown (Raaijmakers and Mazzola, 2012). Microbial genome analysis revealed huge numbers of cryptic antibiotic gene clusters encoding still unknown antibiotics. Antimicrobial metabolites are often considered as the most potent mode of action of microorganisms against competitors allowing antibiotic producing microorganisms competitive advantages in resource-limited environments (Raaijmakers and Mazzola, 2012). Production of antimicrobial metabolites, mostly with broad-spectrum activity, has been reported for biocontrol bacteria belonging to *Agrobacterium*, *Bacillus*, *Pantoea*, *Pseudomonas*, *Serratia*, *Stenotrophomonas*, *Streptomyces*, and many other genera. In *Bacillus*, especially lipopeptides such as iturin, surfactin, and fengycin have been investigated (Ongena and Jacques, 2008), in *Pseudomonas* many antibiotic metabolites such as DAPG, pyrrolnitrin and phenazine have been studied (Raaijmakers and Mazzola, 2012). Many antibiotics are produced only when a microbial population reaches certain thresholds. This quorum-sensing phenomenon is well described for phenazine-producing *Pseudomonas*. Genomic information reveals that also these genera have the potential to produce many still unknown secondary metabolites with possible antimicrobial activity. Also fungal antagonists can produce antimicrobial compounds. For *Trichoderma* and closely related *Clonostachys* (former *Gliocladium*), 6-PAP, gliovirin, gliotoxin, viridin and many more compounds with antimicrobial activity have been investigated (Ghorbanpour et al., 2018). Microorganisms producing antimicrobial metabolites with the potential to interfere with antibiotics in human and veterinary medicine must be excluded from use as MBCAs (Anonymous, 2013a).

The inhibitory effect of secondary metabolites on spore germination or hyphal growth of pathogens can be quantified *in vitro* on nutrient media testing the effects of the antagonistic microorganisms cultured in dual cultures, their metabolites as present in supernatants of cultures of these microorganisms or the purified concentrations of the metabolite. *In vitro* assays are used since the early beginning of scientific research on microbial antagonists, e.g., by Dennis and Webster (1971a, b). Studying inhibitory effects of potential antagonists on agar or in liquid media in dual cultures has several advantages. Assays are fast, resource efficient, highly reproducible and effects are easily to be quantified by measuring colony sizes or percentages of germinated spores. The resulting inhibition zones visualize clearly biocontrol effects and are often used to explain the principles of biocontrol. These advantages may also have led to a bias in biocontrol research. Screening of new antagonists often starts with using *in vitro* assays which are very suitable to detect *in vitro* antagonists which act via antimicrobial metabolites in the artificial environment. This leads to an overestimation of the importance of this mode of action in comparison to other mechanisms which cannot be detected in such *in vitro* assays. As a biased result, in self-fulfilling prophecy, *in vitro* assays may confirm the importance of *in vitro* antibiosis in biocontrol by systematically excluding other modes of action.

The main disadvantage of *in vitro* dual cultures is that production of secondary metabolites depends on nutrient concentration and composition of the chosen medium. Common nutrient media are approximately 100 times richer in nutrients compared to the rhizosphere, and bulk soils are even much less rich in nutrients, supporting even 10–1000 times less bacteria than the rhizosphere (Lugtenberg et al., 2017). Consequently, amounts of secondary metabolites in *in vitro* systems are much higher than reached in natural habitats. Furthermore, agar media or liquid media are ideal for diffusion of the antibiotic compounds which is not the case in habitats such as soil or leaf surfaces. Several studies demonstrated that *in vitro* antagonism does not predict antagonism in complex assays including host plants which simulate the natural habitat situation under controlled or even in field situations (Knudsen et al., 1997). An example is the screening of *Trichoderma* isolates for their potential to control *R. solani*. Köhl (1989) tested 256 isolates belonging to *T. viride*, *T. hamatum*, *T. harzianum*, or *T. koningii* in dual cultures with *R. solani* and in pot experiments with lambs lettuce seeds planted in *R. solani* infested soil. Dual cultures on yeast dextrose agar revealed 192 antagonistic isolates. For these isolates, the average efficacy in reduction of damping off in the pot experiments was 61.2%. For the remaining 64 isolates, showing no *in vitro* antagonism, the average efficacy in pot experiments was similar with 59.7%. This example demonstrates that *in vitro* antagonism depends on the chosen conditions and by far does not explain the antagonistic potential of isolates. Also recent transcriptomic studies confirm that *in vitro* produced metabolites may not be expressed or play a minor role *in situ* (Koch et al., 2018).

Antibiosis observed on agar plates historically resulted in the development of pharmaceutical antibiotics. With similar expectations, results of agar plates often are translated to the control of plant pathogens in the field situation with antimicrobial metabolites seen as sole mode of action against competitors. There is very limited information on measured antimicrobial effects of antagonists *in situ* compared to the large number of publications of *in vitro* effects. Transcriptome analyses of microbial activities in soil confirms that antimicrobial metabolites are produced in soil. Raaijmakers and Mazzola (2012) listed results of various authors who quantified different antibiotics produced *in situ* in soils by bacterial strains introduced at high densities. Production of 5 ng to 5 μg per gram of soil or plant tissue were reported depending on experimental conditions, strains used and type of produced antibiotic with exceptional higher values up to 180 μg per gram for a *Bacillus subtilis* isolate. Antibiotic concentration may be higher in certain microniches, but an important fraction of the antibiotics may be bound to the producing cells and may not diffuse in the habitat (Raaijmakers and Mazzola, 2012). Antibiotics are not stable in the soil environment. Arseneault and Filion (2017) report on half-life of antibiotics produced by biocontrol strains in soil ranging between 0.25 and 5 days depending on biocontrol strain, antibiotic and experimental conditions. Such short life spans can be due to microbial decomposition but also to chemical and physical inactivation. Information on *in situ* concentration of antimicrobial metabolites produced by MBCAs against plant disease and their life span is hardly to be quantified and therefore often missing and not included in risk assessments on non-target effects (Mudgal et al., 2013).

Despite the low concentrations, the inhomogeneous distribution and short lifespan of antimicrobial compounds produced by biocontrol strains *in situ*, studies with mutants of biocontrol strains disrupted in specific antibiotic synthesis demonstrated that antibiotic metabolites play an important role in microbial interactions in soil and plant surfaces (Handelsman and Stabb, 1996; Raaijmakers and Mazzola, 2012). There is increasing evidence that antimicrobial metabolites have important functions for the producing microorganisms at subinhibitory concentrations. In other words: such compounds are characterized as being antibiotic because of their effect on microorganisms at high concentration under *in vitro* conditions although their function in the natural habitat is very different at the prevailing lower concentrations. Arseneault and Filion (2017) discuss modulation of gene expression by low antibiotic concentrations instead of inciting of cell death at high concentrations. Antibiotics at low concentrations can be involved in signaling and microbial community interactions, communication with plants, and regulation of biofilm formation. Raaijmakers and Mazzola (2012) discussed a range of functions of antimicrobial metabolites at low concentrations: there is evidence that antimicrobials including lipopetides protect bacteria from grazing by bacteriovorus nematodes such as *Caenorhabditis elegans*. Also volatile antibiotic compounds may play a role in long-distance interactions amongst soil organisms including bacterial predators. Lipopeptides of *Bacillus* and *Pseudomonas* are involved in the surface attachment of bacterial cells and biofilm formation by activating signaling cascades finally resulting in the formation of extracellular matrices which protect microorganisms from adverse environmental stresses. Some antibiotics, especially lipopeptides support the mobility of bacteria, most likely via changing the viscosity of the colonized surfaces. Surface-active antibiotics allow bacteria to move to nutrient rich locations and also change the water dynamics on leaf surfaces which indirectly affects pathogen development. Other groups of antibiotics influence the nutritional status of plants. For example, DAPG-producing *Pseudomonas* upregulates the nitrogen fixation by plant growth-promoting *Azospirillum brasilense*, and redox-active antibiotics support mobilization of limiting nutrients such as manganese and iron.

Screening of new antagonists acting through antimicrobial metabolites needs to address the insights in ecological functioning of such compounds. Efficient antagonists produce antimicrobial metabolites *in situ* in microniches at sufficiently high concentrations to gain advantage over competitors or at low concentration to fulfill various functions like signaling or nutrient mobilizations, thus functions different from antibiosis. As for most other modes of action, the design of adequate bioassays is essential which combine interactions between potential antagonist, pathogen, plant and are conducted under representative environmental conditions regarding soil environment and microclimate. The often applied *in vitro* screening by far does not mimic the real conditions under which antagonists should be active. However, screening under *in vitro* conditions for strong producers of potential antimicrobial compounds is the first method if the exploitation of the metabolites is envisaged. Antimicrobial metabolites can be produced by selected isolates of antagonistic bacteria or fungi in bioreactors in fermentation processes optimized for high yield of the preferred metabolite. Commercial biological control products may contain microbial metabolites as active ingredient together with the producing microbial antagonist so that after application the direct effect of the metabolite is combined with the potential production of additional metabolite *in situ*. Other products may contain only the produced metabolites, possibly in combination with remains of dead cells of the producing antagonist. Such a use of microbial metabolites is strictly speaking outside the scientific definition of biological control which is defined as the use of living beneficial organisms to suppress populations of plant pathogens (Heimpel and Mills, 2017), but in a broader definition, use of metabolites is also considered as biological control (Glare et al., 2012).

Several reports demonstrate variability within pathogen populations in their sensitivity to antimicrobial secondary metabolites. Selected isolates of *Pseudomonas* spp. produce DAPG with antimicrobial activity against several plant pathogens. A high diversity in sensitivity to DAPG between isolates for *Gaeumannomyces graminis* var. *tritici* has been reported by Mazzola et al. (1995) and for *B. cinerea* by Schouten et al. (2008). Isolates of *B. cinerea* also differ in sensitivity to pyrrolnitrin (Ajouz et al., 2011). These examples indicate that selection pressure by broad use of biological control agents with a single antimicrobial secondary metabolite as mode of action may result in the selection of less sensitive pathogen strains so that the efficacy of the MBCA will not be durable. For *B. cinerea*, a pathogen with high potential to develop resistance against chemical fungicides through adaptation, adaptation to antimicrobial compounds produced by MBCAs has been found (Li and Leifert, 1994). A similar adaptation to pyrrolnitrin, produced by *P. chlororaphis*, was developed by strains of *B. cinerea* in *in vitro* assays with increasing concentrations of the antimicrobial compound in agar growth media (Ajouz et al., 2010). Interestingly, the build-up of resistance resulted in reduced fitness of the strains so that such strains will not persist in absence of selection pressure by pyrrolnitrin. Pathogen strains with higher resistance against antimicrobial compound produced by MBCAs are able to excrete such compounds, e.g., by ABC transporters, degrade the antimicrobial compounds or interfere with the biosynthesis of the compounds by antagonists (Bardin et al., 2015). Since selection pressure depends on dose and exposure duration, the risk for building up resistance is lower if the antimicrobial compounds are produced by the antagonist *in situ* only during direct interaction with the pathogen, often even at subinhibitory concentrations, compared to situations were formulated antimicrobial compounds produced by antagonists already during fermentation are applied at higher dose to the entire crop.

Risk assessments are required for registration of MBCAs as plant protection products for antimicrobial metabolites which are considered as relevant (Anonymous, 2011). Plant pathogenic microorganisms potentially producing mycotoxins and human and animal pathogens potentially producing neurotoxins are excluded from use in biological control. Other secondary metabolites with proven antimicrobial activity which are produced by MBCAs in bioreactors and applied as formulated bioactive compounds included in the end product in amounts effective in disease control (Glare et al., 2012) are relevant metabolites which need to be assessed for potential toxicological and eco-toxicological risks. In addition to the risk assessment performed for the MBCA, a “chemical” risk assessment may be needed for relevant metabolites if they are stable, active without the microorganism, produced at relevant concentrations and present in the MBCA. If such metabolites potentially are produced *in vitro*, but not present in the MBCA or only at low concentration, they are not relevant for risk assessment (Sachana, 2018). However, for the majority of MBCAs, antimicrobial metabolites are produced at low concentrations *in situ* in microniches with low nutrient availability. Concentrations are subinhibitory if modes of action different from antibiosis are exploited (Raaijmakers and Mazzola, 2012). In other situations, metabolite production may be locally and temporally above a minimal inhibitory concentration resulting in inhibition or killing of the targeted pathogen. Such an antibiosis will be restricted in time because of the short life span of antimicrobial metabolites in the environment. Furthermore, the producing antagonist populations will drop after application (Scheepmaker and van de Kassteele, 2011). There is a continuum of microbial activity including production of a great variety of secondary metabolites in the natural environment. Rough estimation of population densities show that even at the moment of application of a MBCA its contribution to the total microbial activity in a given niche is far below 1% (Koch et al., 2018; Lugtenberg, 2018). Unlimited growth of applied saprophytic microorganisms, often a fear of regulators, will not occur in the environment where saprophytic microbial populations are regulated by competitive exploitation of limited resources. Thus, applications of MBCAs with potential *in situ* production of antimicrobial metabolites will not add relevant toxicological or eco-toxicological risks to the cropping system.

In conclusion, antagonists with antimicrobial metabolites as mode of action can be selected using adequate bioassays if *in situ* production by living antagonists is envisaged or *in vitro* if the application of the formulated metabolites is envisaged (Table 1). They generally have a broad host range and their activity depends on environmental conditions if their antagonistic activity depends on *in situ* production, thus on active growth. The risk for development of resistance against antimicrobial metabolites by pathogens can be considered as low in cases where metabolites are produced *in situ*. In cases where a single formulated microbial metabolite is applied on crops, the risk of development of resistance will be, depending on the genetics of the targeted pathogen and the stability of the metabolite in the environment, comparable to risk for chemical active substances. Because of the low concentrations of *in situ* produced antimicrobial metabolites in microniches with low nutrient availability in combination with the typically short lifespans of the metabolites in the environment and the presence of antimicrobial metabolites produced by indigenous microorganisms, toxicological and eco-toxicological risks can be considered as low. If formulated metabolites are applied, their toxicological and eco-toxicological risks are determined by their toxicological profile, the applied concentration and their stability in the environment (Table 2).

## Life Is More Complex: More Modes of Action and Mixed Modes of Action

The research on mode of action of MBCAs usually focuses on induced resistance and priming, competition, hyperparasitism, and antibiosis, but more modes of action are known. For example, fungal viruses in the family Hypoviridae are used to induce hypovirulence in *Cryphonectria parasitica*, the causing agent of chestnut blight (Milgroom and Cortesi, 2004; Double et al., 2018). Other antagonists act via the inactivation of enzymes involved in pathogen infections (Elad, 2000, see below) or the enzymatic degradation of pathogen structures such as a lectin needed by the rice blast pathogen *Magnaporthe oryzae* for spore attachment on the host leaf surface which can be degraded by a specifically selected isolate of *Chryseobacterium* sp. (Ikeda et al., 2013). It can be expected that employing multi-omics will identify many still undetected ways of interactions between microorganisms. It is also known that secondary metabolites and other compounds produced by MBCAs can act through different modes of action. For example, DAPG can have a direct effect as antimicrobial metabolite against the pathogen but also acts as MAMP (Pieterse et al., 2014). Thus, both antibiosis and induced resistance act simultaneously and an artificial separation between the *in situ* effect of DAPG on a single mode of action is hardly possible. Another example is the production of iron-binding siderophores for nutrient competition with the pathogen that are also recognized by the plants as MAMPs inducing resistance (Höfte and Bakker, 2007).

The systematic discrimination of the modes of action of MBCAs is a scientific exercise to unravel how MBCAs act. This information is important for optimizing the use of MBCAs but also asked for registration where the mode of action has to be indicated (Anonymous, 2013a). However, nature of microbial interactions is more complex and does not fit into such pragmatic categories of scientists, regulators, and risk managers. In many cases where the mode of action intensively has been studied for a single biocontrol strain, results confirm that antagonistic interactions are driven by more than one mode of action. Separation into different modes of action is also not always clear and seems to be artificial. For example, *Trichoderma* spp. produce hydrolytic enzymes that permeabilize and degrade the fungal cell wall as one of the key steps in the successful attack of the fungal hosts (Karlsson et al., 2017). The increased permeability of the cell wall is facilitating the subsequent entry of secondary antimicrobial metabolites.

Isolate T39 of *Trichoderma harzianum*, originally selected for the control of *B. cinerea*, also controls the foliar pathogens *Pseudoperonospora cubensis*, *S. sclerotiorum*, and *Sphaerotheca fusca* (Elad, 2000). Isolates of antagonistic *Trichoderma* spp. are generally known to produce antimicrobial metabolites and to act via hyperparasitism (Harman et al., 2004). Detailed studies on *T. harzianum* T39 revealed that no antimicrobial metabolites are interfering with the targeted pathogens. The isolate is able to produce chitinases but Elad (2000) found no correlation between the ability of this strain or other, non-antagonistic strains of *T. harzianum* with their biocontrol activity. *T. harzianum* T39 produces several proteases *in situ* on bean leaves which restrain enzymes of *B. cinerea*. The proteases reduced the activities of the pathogen enzymes exo- and endopolygalacturonase, pectin methyl esterase, pectate lyase, chitinase, β-1,3-glucanase, and cutinase, that are essential for the pathogen during host infection. In experiments with protease inhibitors the biocontrol effect was fully or partially nullified. The biocontrol effect of *T. harzianum* T39 can thus partly be explained by the production of enzymes which suppress pathogen enzymes. The other proven modes of action of *T. harzianum* T39 were nutrient competition, ISR and locally induced resistance. Elad (2000) concluded that various modes of action are responsible for the control of biotrophic and necrotrophic foliar pathogens by *T. harzianum* T39 and he assumed that multiple mechanisms are also involved in other biocontrol systems, but in most cases only part of the possible mechanisms have been elucidated.

*Pseudozyma flocculosa* is an efficient antagonist of Erysiphales (Bélanger et al., 2012) that does not penetrate powdery mildew cells but cause a rapid cell death. *P. flocculosa* can produce 6-methyl-9-heptadecanoic acid and the glycolipid flocculosin. Since there was no evidence for induced resistance in treated plants and nutrient competition seemed to be unlikely in antagonism against a biotrophic pathogen, it was concluded that antibiosis is the sole mode of action. However, gene expression studies revealed that there was no significant increase in expression of the relevant genes at any time during the antagonistic process so that other modes of action must be responsible (Bélanger et al., 2012). There is now increasing evidence that competition for the micronutrients Zn and Mn plays a role during the dedicated tritrophic interaction: powdery mildew takes up these elements from the host plant and *P. flocculosa* draws these elements then from the pathogen.

Both examples of in depth investigations of the mode of action of MBCAs illustrate that tritrophic interactions between host, pathogen and MBCA are complex and often different from what is initially expected (Elad, 2000; Bélanger et al., 2012). New, rather unexpected (combinations of) mechanisms may be revealed by future analysis of the increasing genomic and transcriptomic information. Current examples are studies on gene expression of *Clonostachys rosea* (Nygren et al., 2018) and the genome analysis of *Metschnikowia fructicola* (Piombo et al., 2018). The examples also illustrate that antagonists evolve a great variety of (combinations of) mechanisms to interact with other microorganism rather than rely on using a “single molecule approach” similar to the approach of using synthetic fungicides. Such a highly regulated *in situ* production of various ubiquitous mechanisms commonly used in the microbial interplay in the environment makes the use of MBCAs a particular safe and sustainable technology.

Because of the ubiquitous character of *in situ* modes of action specific risk assessments are not relevant. Because of the complexity of the cascades of physiological events the indication of the principal (single) mode of action as data requirement of Commission Regulation 283 (Anonymous, 2013a; see Box 1) is impossible.

**Box 1.**

What are the data requirements and the uniform principles concerning the mode of action of the microorganism against plant diseases in the EU?

The most important data requirements related to the mode of action of active substances are set out in Commission Regulation (EU) No. 283/2013 (Anonymous, 2013a).

“*The principal mode of action shall be indicated. (…)”* If *“the micro-organism produces a toxin with a residual effect on the target organism (…), the mode of action of this toxin shall be described.”*

“*If the plant protection action is known to be due to the residual effect of a toxin/metabolite (…), a dossier for the toxin/metabolite has to be submitted (…)*”

*“Any available information on the mechanism (…)” and “(…) the influence of the produced metabolites on the micro-organism’s mode of action shall be provided.*”

The data requirements for plant protection products (preparations) are set out in Commission Regulation (EU) No. 284/2013 (Anonymous, 2013b).

“(…) the pest controlling action (fungitoxic, fungistatic action, nutrient competition, etc.) must be stated.

It must also be stated whether or not the product is translocated in plants and, where relevant, if such translocation is apoplastic, symplastic or both.”

The uniform principles for evaluation and authorisation of plant protection products are set out in Commission Regulation (EU) No. 546/2011 (Anonymous, 2011).

The micro-organism in the plant protection product should ideally function as a cell factory working directly on the spot where the target organism is harmful. (…)

“Micro-organisms may produce a range of different metabolites (e.g., bacterial toxins or mycotoxins) (…)” that “(…) may be involved in the mode of action of the plant protection product. The characterization and identification of relevant metabolites must be assessed and the toxicity of these metabolites must be addressed. (…)

The mode of action of the micro-organism shall be evaluated in as much detail as appropriate. The possible role of metabolites/toxins for the mode of action shall be evaluated and (…) the minimal effective concentration (…) shall be established. (…) Aspects to be considered in the evaluation, are:

(a) antibiosis;

(b) induction of plant resistance;

(c) interference with the virulence of a pathogenic target organism;

(d) endophytic growth;

(e) root colonization;

(f) competition of ecological niche (e.g., nutrients, habitats);

(g) parasitization;

(h) invertebrate pathogenicity.”

Mode of action is taken into account at evaluation of the degree of adverse effects on the treated crop, operator exposure, viable residues, fate, and behavior in the environment and at risk assessment of birds, mammals, aquatic organisms, bees, arthropods other than bees and earthworms and nitrogen and carbon mineralization in the soil.

## Novel Biocontrol Approaches and Mode of Action

Microbial biological control agents interact with the plant, the targeted pathogen and the resident microflora. Studies on the interactions with the resident microflora have been hampered in the past because of limitations of available methods. This changed drastically with arrival of Next Generation Sequencing (NGS) methods such as metagenomics and metatranscriptomics allowing to identify the composition and functions of the microbiome (Massart et al., 2015a, 2015b). Further steps by adding information of metametabolomics and signalomics (Mhlongo et al., 2018) will complete the picture on interactions between introduced MBCAs and resident microbiota. As a result, a holistic in depth understanding on MBCA-microbiota interactions will support better timing, formulation and application of MBCAs and prevent failures. It is expected that three new developments will have significant impact on biological control of plant diseases. First, functional analysis will allow a “prebiotic approach” (Massart et al., 2015a). Application of specific compounds or complex substrates will modulate indigenous microbiota compositions with the aim to enhance microbial suppression of plant pathogens (Mazzola and Freilich, 2017). Such a manipulation of resident microbiota toward disease suppression may be comparable to conservation biological control applied in insect pest control, e.g., via improving nutrient availability for beneficial insect populations by planting flower strips. Simple or complex substrates applied for such a prebiotic approach may not be considered as plant protection products. A second approach will be the selection and application of “helper” strains (Massart et al., 2015a) which have no biocontrol properties on their own but support MBCAs in establishment, survival and antagonistic activity *in situ*. A third expectation is that core microbiomes will be designed (Gopal et al., 2013; Massart et al., 2015a; Syed Ab Rahman et al., 2018) consisting of different strains of biological control species combining various modes of action. Gopal et al. (2013) stated that the transfer of tailor-made core-microbiomes will become a viable strategy for plant disease management.

The ecological considerations supporting the idea of assembled consortia are sound (Table 1). However, practical considerations may hamper their introduction. Validation and optimization of *in silico*-designed consortia under ranges of relevant environmental conditions will be complex and will need substantial resources. In a commercial setting, development of mass production, down streaming and storage procedures separately for each individual consortium member will need substantially more resources and investments compared to production of single strain MBCAs (Table 1). Registration of assembled consortia as plant protection products will add further difficulties. Regulations in the EU demand the risk assessment of each active ingredient before the product can be registered. In case of assembled consortia, costs will thus increase substantially. In this context, strategies to develop helper strains or to shape the indigenous microbiota may clearly have advantages above the use of assembled core consortia (Table 2). On the other hand, an adapted legislation for novel disease control systems would benefit society as a whole as well as the environment.

## Synthesis and Future

Microbial biological control agents use a broad arsenal of modes of action which are used wherever microorganisms interact, communicate, and regulate their co-existence between microbial cells and between microorganisms and plants. The exploitation of different modes of action has different advantages and disadvantages in relation to the development of commercial MBCAs by industries and their practical use by growers (Table 1), but also regarding the perception of possible toxicological and ecotoxicological risks for producers, users, consumers, and the environment (Table 2). Studies on mode of action of well-documented antagonists show that antagonism generally is not based on a single action of a certain mode of action, but on a sequence of events with the use of different modes of action over time. During such cascades of physiological events signals often are the result of the earlier used modes of action, e.g., cell wall degradation products after use of CWDEs (Karlsson et al., 2017).

For the development of specific biocontrol products, certain modes of action may be preferred. In such cases, screening of new MBCAs can be very focused, e.g., on selection of suitable MAMPS inducing resistance or priming the host plant, utilization of a specific nutrient element or substrates, or the selection for a potential antibiotic metabolite. This screening strategy may be powerful if new strains are being selected superior to an existing, well characterized antagonist or for further strain improvement within an existing antagonist strain. However, in most other cases, selection procedures should be preferred that allow the selection of new combinations of known and still unknown modes of action which are produced directly at the site of interaction. This view on preferable biocontrol mechanisms is expressed also in Commission Regulation (EU) 546/2011 (Anonymous, 2011) which states “*that micro-organism in the plant protection product should ideally function as a cell factory working directly on the spot where the target organism is harmful*.” For this objective, the overall effect on pathogen and disease development has to be assessed rather than the expression of a single expected main mechanism of action. The key challenge for screening projects is thus the development of suitable robust bioassays which combine the interactions between pathogen, host, and antagonist under controlled conditions. Depending on disease characteristics and host, the design of such assays can be troublesome and challenging, especially if “difficult biology” has to be combined with the cost-effective testing of large numbers of candidate antagonists. Attractive alternative routes via *in vitro* tests should not be used to avoid biased selection with emphasis on one mode of action, thus excluding many other powerful modes of action or combination thereof, which may be even ineffective at all if evaluated on their own.

The efficacy of biological control agents against plant diseases may not be durable because pathogen populations may develop resistance comparable to the frequently observed build-up of resistance against chemical fungicides with a single mode of action. Important factors for an erosion of effectiveness are variation in susceptibility to the mode of action within the pathogen population, selection pressure resulting in shifts within pathogen populations toward less susceptible strains and the fitness of the selected strains in the environment under conditions without selection pressure (Bardin et al., 2015). For the choice of a certain mode of action, the kind of selection pressure needs to be considered in respect to the pathogen’s evolutionary potential which determine the ability to adapt to the selection pressure via selection within a population with sufficient variation in susceptibility. Variation in susceptibility of pathogens has been found for some pathogens such as *S. sclerotiorum* and *G. graminis* var. *tritici* (Mazzola et al., 1995; Schouten et al., 2008; Nicot et al., 2016). However, development of resistance has not reported yet for commercially used biological control products for control of plant diseases (Nicot et al., 2011). The risk of resistance development in MBCAs used in sustainable IPM systems is also low because IPM combines a variety of measures to prevent damage by diseases without relying on a single control method.

The build-up of resistance is a serious problem in single molecule-single mode of action chemical fungicides which shorten their economic life span. For MBCAs the principle modes of action exhibit much less selection pressure on pathogens additional to the always present selection pressure during natural competitive interactions of organisms. Furthermore, it is common that a combination of different modes of action are active and each mode of action is based on multiple actors, e.g., different CWDEs, a set of different MAMPs or different antimicrobial metabolites with sometimes very different signaling functions. For MBCAs it thus can be concluded that build-up of resistance is much less likely compared to the build-up of resistance against chemical plant protection products. Only exceptional uses of MBCAs such as the use of *in vitro* produced highly concentrated and purified secondary metabolites or the use of genetically modified MBCAs with extraordinarily high expression of a single antimicrobial metabolite may result in selection pressures comparable to single site fungicides.

Knowledge of the mode of action of the microorganisms is required and has also to be considered in the context of other potential risks before a MBCA can be approved for use as plant protection product. Risk assessments of MBCAs are regulated in the EU by Regulation (EC) No. 1107/2009 (Anonymous, 2009) and by Commission Regulation (EC) No. 546/2011 (Anonymous, 2011) regarding the uniform principles for evaluation and authorisation of plant protection products containing microorganisms, by Commission Regulation (EU) No. 283/2013 in its Part B on microorganisms including viruses (Anonymous, 2013a) setting out the data requirements for active substances and by Commission Regulation (EU) No. 284/2013 in its Part B on microorganisms including viruses (Anonymous, 2013b) setting out the data requirements for plant protection products (Box 1).

The regulations focus strongly on the possible risks of secondary metabolites and toxins potentially produced by microorganisms. Several groups of fungi are known to produce mycotoxins, several groups of bacteria are known to produce toxins including the botulinum-neurotoxin (BoNT). Microorganisms producing such mycotoxins or toxins in relevant amounts are excluded from the use in biological control because of the potential risks for humans and animals. MBCAs may produce other secondary metabolites as sole mode of action, or – in the majority of cases – as component of a cascade of different secondary metabolites in combination or alternation with other metabolites such as CWDEs or MAMPS. The function of the produced metabolites often is not antibiosis but to fulfill other functions including signaling at subinhibitory concentrations. Secondary metabolite production is highly regulated and restricted to micro niches and in time. Such metabolites are rapidly degraded and thus have short life spans in the environment.

Only for MBCAs which produce potential antimicrobial metabolites *in vitro* or during the mass production fermentation process and contain such metabolites in the formulated end product at effective concentration, thorough risk assessment is indicated and the minimal effective concentration against the target and representative non-target organisms can be established. However, in all other cases, such metabolites are not relevant for a risk assessment. Furthermore, reliable quantification of temporal metabolite concentrations in microniches in the *in situ* situation can hardly to be achieved. The perception of risks caused by antimicrobial metabolites in biological control may be more a result of the broad use of *in vitro* studies on antibiosis in biocontrol research rather than the result of studies on on-site production of such metabolites in the environment. *In vitro* antagonism can easily be visualized through inhibition zones on culture media. The similar method is used for the screening of pharmaceutical antibiotics that aims at the development of products containing single molecules for medical treatments. Communication on biocontrol research based on *in vitro* assays, showing inhibition zones, may create a wrong view on the nature of biocontrol control resulting in the fear of the use of antibiotics in crop protection. Since results of *in vitro* assays generally do not correlate with results obtained in bioassays or with crops (Koch et al., 2018), there are no reasons to rely on such artificial systems in studies on antagonist screenings and in research on the function of microbiomes. Biocontrol research unraveling the mechanisms in the much more complex *in situ* situations may reduce the unjustified fears for microbial metabolites produced by MBCAs.

In conclusion, MBCAs are functioning directly on the spot where the targeted organism is harmful (Anonymous, 2011), generally combining different modes of action to highly regulated cascades of events. Current thinking on how to consider the mode of action during the risk assessment and registration procedure of MBCAs focuses on a single mode of action and potential risks of *in vitro* produced metabolites, very similar to the risk assessment of synthetic fungicides with a single compound as active ingredient. A rethinking is needed considering that the effectiveness of MBCAs in most cases is based on natural, complex, highly regulated interactions between microbial cells and plants on site but are not the results of a single action of a single metabolite. Toxicological and ecotoxicological risks of such complex processes of interaction can be considered as very low. Moreover, humans and other organisms have been and still are exposed to such processes in evolutionary terms and adverse effects are not known. Since an antimicrobial action of a single metabolite is not relevant in many cases, the existing EU regulations may require such a rethinking in the registration of MBCAs as long as antimicrobial metabolites are not present in the formulated MBCA at relevant concentrations.

## Summary Points

1. Microbial biological control agents use a great variety of mechanisms to protect plants from pathogens.
2. Important modes of action strengthen the resistance of the plant, e.g., induced resistance or priming, or modulate the local growth conditions for pathogen development, e.g., nutrient competition, but do not interfere directly with the pathogen.
3. Hyperparasitism and secondary metabolites are directly affecting the targeted pathogen via highly regulated cascades of physiological events but not by a single constitutively produced metabolite.
4. Secondary metabolites produced *in vitro* may have antimicrobial activity at high concentration but low amounts are produced *in situ* very locally during interaction and metabolites have short life spans, often with functions such as signaling, very different from antibiosis.
5. During the cascades of events a range of different compounds with different modes of action are used to outcompete the pathogen. Such events of signaling and interaction are common wherever microorganisms interact.
6. The highly regulated *in situ* production of ubiquitous mechanisms commonly used in the microbial interplay makes the use of MBCAs a safe and sustainable technology.
7. *In situ* produced compounds such as MAMPs, enzymes or secondary metabolites are not relevant for risk assessments so that detailed toxicology and ecotoxicological studies of these compounds are not relevant, and should not be required.
8. The fear of antimicrobial metabolites produced by MBCAs after their release is not based on real risks but fed by the wrong perception on how biocontrol acts if studied under *in vitro* conditions.
9. If antimicrobial metabolites are the active ingredient in the formulation of the biocontrol product, risk assessment of such metabolites is relevant.

## Future Issues

1. Better screening assays for finding the next generation of MBCAs are needed to measure the overall effect of the interplay of different modes of action.
2. Multi-omics will help to further understand the complex events during microbial interactions in the environment.
3. Current EU regulations on registration of MBCAs should allow a science-based differentiation between the majority of compounds involved in modes of action to be considered as safe and not relevant for detailed risk assessment and the limited number of cases relevant for risk assessments where secondary metabolites are present as active ingredients in MBCAs formulations at high concentrations.

## Author Contributions

JK conceived and designed the research. JK, RK, and WR contributed to the manuscript and revised it critically for important intellectual content. All authors approved the final version of the manuscript.

## Conflict of Interest Statement

RK was employed by company Linge Agroconsultancy b.v. and WR was employed by company Koppert Biological Systems. JK declares no competing interests.
